# Flexible Dye-Sensitized Solar Cell Based on Vertical ZnO Nanowire Arrays

**DOI:** 10.1007/s11671-010-9804-x

**Published:** 2010-09-26

**Authors:** Sheng Chu, Dongdong Li, Pai-Chun Chang, Jia G Lu

**Affiliations:** 1 Department of Physics and Department of Electrical Engineering, University of Southern California, Los Angeles, CA 90089-0484, USA; 2 Division of Energy and Environmental Research, Shanghai Advanced Research Institute, Chinese Academy of Sciences, Shanghai, 201203, China

**Keywords:** ZnO nanowires, Vertical alignment, Peel off, Dye-sensitized solar cells, Flexible electronics

## Abstract

Flexible dye-sensitized solar cells are fabricated using vertically aligned ZnO nanowire arrays that are transferred onto ITO-coated poly(ethylene terephthalate) substrates using a simple peel-off process. The solar cells demonstrate an energy conversion efficiency of 0.44% with good bending tolerance. This technique paves a new route for building large-scale cost-effective flexible photovoltaic and optoelectronic devices.

## Introduction

Flexible solar cells have attracted tremendous interest because of its potential for wearable electronics and other versatile applications [1]. Dye-sensitized solar cell (DSSC) is well known as one competitive alternative to conventional inorganic photovoltaic cells due to its simplicity, low cost, and good power conversion efficiency [2]. Typically, the photoanodes of flexible DSSCs consist of titanium oxide nanocrystals on a plastic substrate that are produced by utilizing low-temperature processes, such as sintering [3], mechanical pressing [4], hydrothermal crystallization [5], electrophoretic deposition [6], microwave irradiation [7], or film transfer [8]. However, low-temperature treatments usually result in poor material crystallinity and fragile characteristic, thus limit the potential of DSSCs as flexible electronics. Recently, significant progress in photovoltaics has been achieved based on one-dimensional materials owing to the improved crystalline quality, efficient charge separation/transport process, and mechanical flexibility [9-15].

ZnO, an *n*-type semiconductor, is known for its high electric conduction and carrier mobility [16]. ZnO has demonstrated as a good photoanode in DSSCs since its energy band structure is similar to that of TiO_2 _[17,18]. Additionally, the syntheses of one-dimensional ZnO materials with controllable structures have been well developed by using either vapor or solution phase growth methods [11,19]. In this work, vertically aligned ZnO nanowire arrays with high aspect ratio are synthesized by vapor phase growth. A robust peel-off technique is employed to transfer vertically aligned nanowire arrays onto indium tin oxide (ITO)-coated poly(ethylene terephthalate) (PET) flexible substrate. Bendable ZnO nanowire-based DSSCs are then assembled following polymer packaging with nanowires as electrodes. The fabricated device shows good tolerance under strong mechanical bending.

## Experimental Methods

Aligned ZnO nanowire arrays are synthesized via seed growth scheme [20]. A ~500 nm ZnO seed layer is first deposited onto an *n*-type Si (100) substrate (resistivity in the range of 1–10 Ω cm) in a molecular beam epitaxy system. Pure zinc powder is then placed at the center of a horizontal tube furnace for the ZnO nanowire array growth. Oxygen carried by Ar (1:1000) is kept flowing during the synthesis. The typical growth and characterization of ZnO nanowires can be found in our previous reports [19,21,22].

Polydimethylsiloxane (PDMS) solution, made by mixing the base and curing agent (10:1 w/w) (Sylgard 184), is diluted (5:1 w/w) by a hexamethylcyclotrisiloxane solution in methylene chloride (Alfa Aesar). The solution is subsequently dropped onto the nanowire film and spun at a rate of 2,000 rpm for 1 min to allow uniform distribution of a thin PDMS layer at the bottom of ZnO nanowire arrays. After a heat treatment at 150°C for 1 h, the PDMS layer is cured at the bottom of the ZnO nanowire array forming a matrix. This PDMS matrix together with the embedded ZnO wires can be then removed from the wafer mechanically. The free-standing nanowire/PDMS film is then mounted onto an ITO/PET substrate using silver paste for subsequent device fabrication process.

Before the DSSC device packaging, the prepared ZnO/PDMS/Ag/ITO/PET film is dipped into a 0.3 mM N-3 dye (*cis*-Bis(isothiocyanato)bis(2,2'-bipyridyl-4,4'-dicarboxylato)ruthenium(II)) in *t*-butanol/acetonitrile solution (1:1 volume ratio) for 24 h of dye loading. A parafilm is used as a spacer layer between the nanowire array and a Pt (2 nm)-coated ITO/PET counter electrode. The electrolyte containing 0.5 M LiI, 0.05 M I_2_, 0.5 M *tert*-butylpyridine in acetonitrile and valeronitrile (1:1 volume ratio) is filled in the space between ZnO nanowire and the Pt/ITO counter electrode to construct the solar cell.

## Results and Discussion

**Figure 1:**
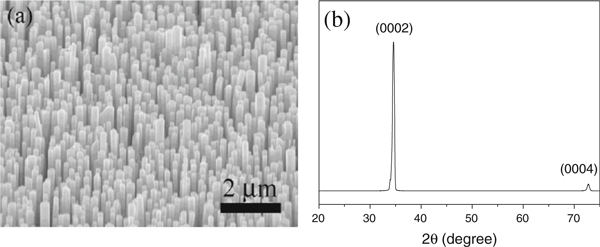
**a A SEM image and b XRD pattern of vertically aligned ZnO nanowire array grown on the ZnO seed film**.

Single crystalline wurtzite ZnO film with preferential direction along the *c*-axis has been reported [23]. The seed film facilitates the epitaxial growth of nanowires along *c*-axis due to the perfect match of lattice parameters [24]. Figure 1a is a scanning electron microscopy (SEM) image of ZnO nanowire arrays, showing hexagonal cross-section facets grown from the seed substrate. The average length of nanowire is ~15 μm, while the diameter falls in the range of 200–400 nm. Figure 1b shows the X-ray diffraction pattern (XRD) of ZnO nanowire arrays. The observed two peaks can be indexed to (0002) and (0004) crystal planes (JCPDS No. 361451).

**Figure 2:**
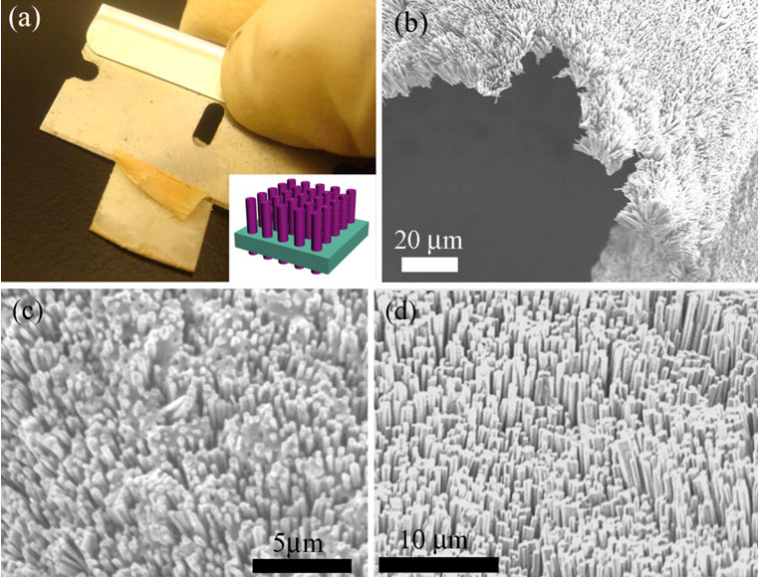
**a A photograph showing razor blade peel-off process**. *Inset*: a schematic illustration of ZnO nanowire array embedded in the PDMS matrix. SEM images of **b** the peeled-off ZnO/PDMS film with good bending ability. **c** flip-over *bottom view* of ZnO/PDMS film showing exposed ZnO tails, and **d** the *top view* of ZnO/PDMS film transferred onto ITO/PET substrate.

Recently, Plass et al. [25] presented a simple method to peel off oriented Si nanowire arrays from rigid substrate by using a polymer film matrix. Here, a similar peeling-off technique is carried out to transfer vertically aligned ZnO nanowire arrays onto indium tin oxide (ITO)-coated poly(ethylene terephthalate) (PET) flexible substrate. Figure 2a illustrates the removal process of nanowire/PDMS matrix by employing a razor blade. Normally the as-peeled film covers the whole substrate area (~1.5 cm^2^ in this work). This process yields a large area single crystalline ZnO nanowire array embedded in a transparent, yet mechanically and chemically robust PDMS sheet (as illustrated in the inset schematic of Figure 2a).

Figure 2b is the SEM image of ZnO nanowire/PDMS film while the nanowire remains good directional alignment. The bent film indicates the excellent mechanical flexibility through utilizing PDMS polymer matrix. The bottom view of ZnO/PDMS film (Figure 2c) shows that the ZnO nanowires are interconnected by PDMS whereas the exposed wire tails will be implemented as the contact electrodes. Figure 2d shows the top view of ZnO/PDMS composite in which the nanowires maintain good vertical alignment after transferring onto ITO substrate.

**Figure 3:**
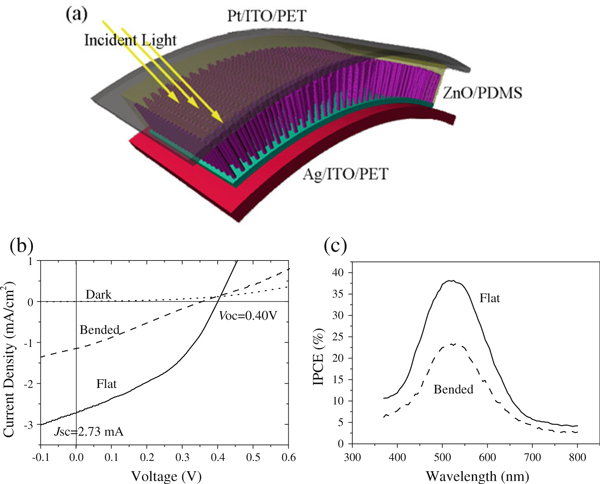
**a A schematic view of the flexible DSSC**. **b** Photocurrent density–photovoltage (*J*–*V*) curves of the ZnO nanowire array DSSC without illumination (*dotted line*), under illumination without bending (*solid line*), and with bending (*dash line*) with a bending radius ~5 mm. **c** The quantum efficiency of ZnO nanowire array DSSC with/without mechanical deformation.

Figure 3a illustrates a schematic view of the completed ZnO nanowire array-based flexible DSSC. The photovoltaic properties are characterized under a simulated AM1.5G illumination (standard 100 mW/cm^2^) where a light radiation is introduced to the active dye from the side of the Pt/ITO-PET transparent electrode. Figure 3b shows the photocurrent density versus photovoltage (*J*–*V*) curves of the device. Without any mechanical bending (solid curve), the device exhibits a short-circuit current density (*J*_SC_) of 2.73 mA/cm^2^, open-circuit voltage (*V*_OC_) of 0.40 V, and a filling factor (*FF*) of 40%. The corresponding power conversion efficiency (*η*) is calculated to be 0.44%, comparable to recently reported ZnO nanowire-based DSSC [26,27]. The dashed curve shown in Figure 3b is the device under a strong bending (bending radius ~5 mm), resulting in a reduced *J*_SC_ (1.15 mA/cm^2^), *FF* (26%), and an efficiency of 0.11%, whereas *V*_OC_ remains the same. Although the performance of non-bending solar cell is stable, the *J*–*V* curves vary with different forces and angels. The reduction in efficiency upon bending is attributed to the loss of light absorption and induced mechanical defects. Bending will naturally deteriorate the non-perfect contact between Ag paste and ZnO, as well as of the top contact, thus increasing the series resistance in the solar cell. Furthermore, the Pt film on ITO substrate is not continuous and could likely be removed upon bending, subsequently decreasing the efficiency of redox reaction in the electrolyte.

The incident photon-to-current conversion efficiency (IPCE) value, referred as the quantum efficiency (QE), is the ratio of the observed photogenerated charge carriers to the incident photon flux, uncorrected with reflective loss for optical excitation through the conducting transparent electrode. Figure 3c plots the quantum efficiency of the flexible ZnO nanowire DSCC with and without bending. The maximum efficiency of 38% is achieved at 515 nm, which is comparable to the competitive solar cells built from ZnO nanoporous film on rigid substrates [28]. A tail in the UV region close to ZnO band gap is observed owing to the light harvesting directly from ZnO. The QE of the device under bending shows a reduced value (~23%) with the same peak position at 515 nm. The degraded performance is due to the loss of light absorption and the bending induced mechanical defects.

## Conclusions

Large scale single crystalline ZnO nanowire arrays have been successfully transferred onto flexible substrates maintaining vertical orientation by employing PDMS matrix. This technique offers a simple and robust way to realize the application of vertically aligned nanowire array on arbitrary substrates via low-temperature process. The ZnO nanowire array-based DSSCs constructed on flexible ITO/PET substrate demonstrate reasonable energy conversion efficiency and good mechanical bending tolerance. However, the performance of the presented DSSC device has deficiencies. The current light illumination direction from the Pt-coated ITO/PET counter electrode limits the cell performance since photons will be partially reflected by Pt coating. Second, a shortcoming of the DSSC is the usage of silver paste that does not provide good electrical contact between the nanowire array and ITO electrode substrate. More technical enhancement is required to improve the device functionality.
